# Supplementary material for: Radiographic evaluation of patellar tendon length following corrective surgical procedures for medial patellar luxation in dogs
Source: PLoS One. 2020 Sep 4;15(9):e0238598. doi: 10.1371/journal.pone.0238598 (PMC7473547; doi:10.1371/journal.pone.0238598)
Supplement: Attachment — Submitted filename: Response to Reviewers.docx [file pone.0238598.s002.docx]

Dear PLOS ONE Editors and Reviewers,

Thank you for the critiques and suggestions for our manuscript. The majority of the recommendations made have added clarification and quality to the discussion of our study. We feel that the revised manuscripts have adequately addressed the concerns raised during the review process. Please see review the following rebuttal letter that addresses suggestions made. We hope that the revised manuscript is suitable for publication and look forward to hearing from you in due course.

Best, Kevin de Moya

1. Please ensure manuscript meets PLOS ONE’s style requirements including those for file naming

- The manuscript formatting has been updated including references and requirements made for file naming.

2. Please include your amended funding statements within your cover letter.

- See updated cover letter.

3. Please amend the manuscript submission data (via Edit Submission) to include author Stanley Kim.

- See amended manuscript.

4. Please include captions for your Supporting Information files at the end of your manuscript, and update any in-text citations to match accordingly.

- See amended manuscript.

5. Review comments to Author (Reviewer #1)

- Comment- Correct “patellar” to “patella” (line 39).
- Response- As suggested, this correction has been made (line 39).
- Comment- Add more references (line 47-50).
- Response- As suggested, additional references have been added to support the statements made (line 46-50).
- Comment- Confusing, reporting some info as on line 40-42 (line 50-52).
- Response- This sentence has been deleted.
- Comment- This is probably non-relevant information. The paper deals with MPL and any other affection of the stifle lead to exclusion. I would erase it (line 52-54).
- Response- As suggested, this sentence has been deleted.
- Comment- Please discuss, for a better understanding of the Reader, why this can happen (line 54-56).
- Response- As suggested, an additional sentence has been added to further clarify the statements made (line 51-53).
- Comment- Please change “would” to “might”. Your hypothesis is correct from my point of view; however have you considered that some dogs, especially some small breed, can have concomitant MPL and patella baja, and in this case the elongated patellar tendon should improve the tracking and not increase the risk of luxation. Maybe you should specify that the hypothesis is limited to dog with a relatively normal position of the patella (line 62-63).
- Response- As suggested, an addition to the hypothesis was added clarifying that our hypotheses apply to dogs with relatively normal patella positions (57-60).
- Comment- Is 2-3 months an appropriate follow up? Patellar tendon stretching and elongation might take longer, also some complications as reluxation might happen later in time (line 71).
- Response- 2-3 months is the last radiographic follow up time point before animals are discharged to resume normal activity, assuming no complications occurred. Although we suspect that patellar tendon lengthening can occur past this time point, we do not enough cases with radiographic follow up past this time point.
- Comment- Please remove “from medical records”; correct “include” to “included”; please remove “comorbidities” because you stated previously your inclusion/exclusion criteria; change “surgical procedure(s) and post-operative complications” (line 71-73).
- Response- The statement has been updated to reflect suggestions made (line 69-71).
- Comment- Please change “Surgical corrective procedures performed include” to “Surgical treatment included” (line 73).
- Response- The statement has been updated to reflect suggestions made (line 70-71).
- Comment- The reference to the Figure should be moved at the end of the sentence that describes the methodology of measurement (line 78).
- Response- The reference to Figure 1 has been moved to the end of the sentence that describes the measurement methodology (line 83).
- Comment- I am not convinced of the first sentence, it does not sound well. I would suggest to change this part as follow “All measurements were performed on a standard mediolateral radiographic projection of the stifle by using a dedicated PACS workstation using DICOM viewing software (Merge Healthcare Inc, Chicago, Illinois)- (line 77-80).
- Response- The statement has been updated to reflect suggestions made (line 75-77).
- Comment- Can you mention the angle between femur and tibia? Do you think that limb positioning (in this case the degree of flexion) may affect measurement and result? (for replicating your study it is important to give to the Reader all the relevant information)- (line 80-81).
- Response- An additional statement has been added with proper reference justifying why the degree of stifle flexion was not included in the study (92-94).
- Comment- Vertical position of the patella in dogs has been previously reported and several methods have been described (for example Mostafa et al. and Johnson et al. described two different measurements). Can you please add a reference to your methodology in order to help the Reader to understand that not all the published studies are comparable between each other? (line 82-86).
- Response- The statement has been updated to reflect that the measurements made are consistent with those of previous studies (83-85).
- Comment- Regarding landmarks, have you noticed any implant interference in identifying the correct landmark? The accuracy of measurement might be discussed (line 88).
- Response- An additional statement has been added describing the methodology used in cases where there was implant interference with bony landmarks (89-91).
- Comment- “On a total of 242 stifles surgically repaired, only 50 cases met the inclusion criteria”. Maybe sounds better (line 110).
- Response- The statement has been updated to reflect suggestions made (line 117).
- Comment- “The majority of cases were excluded due to inadequate radiographic follow up”. I think you can delete this sentence since you stated previously your inclusion/exclusion criteria (line 111).
- Response- As suggested, this statement has been deleted.
- Comment- Here I would mention a sentence that I suggested to remove in the introduction regarding the reported changings following TPLO procedure. I would start the discussion stating that the patellar tendon is subjected to changings and that some pathologies as well treatments can affect its length. Try to find further references (also check human literature) to strengthen this statement because this is the pillar of the paper (line 134).
- Response: This paragraph has been updated to reflect both veterinary and human studies that investigate changes in patellar tendon length as an introduction to our discussion. The veterinary studies that were previous mentioned in the introduction were moved to this section (141-153).
- Comment- Please check the literature, there are papers that provide some information that are in contrast with studies you cited. It is important in the discussion review all the available literature to find evidence. “Additionally, patella alta has previously been proposed as predisposing factor to post-operative recurrence of luxation (Johnson 2006)”, but patella alta has also been observed in healthy dogs that did not exhibit orthopaedic problems in the stifle joints (Łojszczyk-Szczepaniak, A., Silmanowicz, P., Komsta, R. et al. Determination of reference values and frequency of occurrence of patella alta in German shepherd dogs: a retrospective study. Acta Vet Scand 59, 36 (2017)). If you do not clarify this statements it sounds like that if the patella is alta you can have reluxation. I believe this complication is more related to other abnormalities rather than a patella higher than normal (line 139-142).
- Response- As suggested, the statement has been updated with further clarification and additional references (line 149-153).
- Comment- Add more references (line 160).
- Response- As suggested, this paragraph has been updated with additional references (line 176).
- Comment- This is probably one of the most interesting part of the paper. You must answer this question “Why grade 3-4 MPL have not significantly different ratios?”. This is probably due to the pre-existing elongation of the tendon that is subjected to abnormal tension during growth or normal physical activity (Line 161-167).
- Response- As suggested, this paragraph has been updated to give a proposal of why grade 3-4 MPL may not have significantly different ratios (181-183).

6. Review comments to Author (Reviewer #2)

- Comment- Please add the name of the second co-author (all co-authors) at the beginning, in the “order of authors”.
- Response- The name of the second co-author has been added in the “order of authors” section.
- Comment- According to the journal guidelines, the references should be cited as the numbers in brackets. They should be numbered in the order they appear in the text. Please revise your citations.
- Response- The references have been updated and are cited as numbers within brackets and are in the order they appear in the text.
- Comment- According to the journal guidelines, the abstracts should not include abbreviations if it is possible.
- Response- The abstract has been updated and does not include any abbreviations.
- Comment- Please revise the word “Patellar alta” to “Patella alta” (line 39).
- Response- As suggested, the statement has been updated to reflect suggestions made (line 39).
- Comment- You did not talk about LPL in your study. Please explain if you excluded the dogs with LPL from your study. If LPL affected dogs were not excluded please explain the dogs with MPL and LPL. Is there any relationship between the dogs with bidirectional patellar luxation and post-operative patellar tendon length? (line 68-69).
- Response- A statement has been added to the Methods section that clarifies dogs with lateral patellar luxation were excluded from the study (line 66-67).
- Comment- Please talk about the muscle contracture in your study and if the radiographs were taken under general anesthesia.
- Response- As suggested, an additional statement has been added regarding anesthesia and sedation for radiographs (line 77-78).
- Comment- Please revise the sentence "the distance from the distal most aspect of the patellar to the proximal aspect of the tibial tuberosity" as "the distance from the most distal aspect of the patella to the most proximal aspect of the tibial tuberosity" (line 84-85).
- Response: The statements have been updated to reflect suggestions made (line 80-83).
- Comment- Please revise the sentence "Patella tendon: patellar ratio" as "Patellar tendon length: patellar length ratio" (line 93-94).
- Response- The statement has been updated to reflect suggestions made (line 86-87).
- Comment- Please include the name and version of the used statistical software and calculation methods (name of the tests) in the material and methods.
- Response- A statement has been added to reflect the suggestions made (line 114-115).
- Comment- Please discuss the included dog breeds in your study. You only mentioned in the discussion that you had small to large breed dogs; it would not be out of interest if you could explain the frequency of each breed.
- Response- Dogs were categorized based on weight (<10kg vs >10kg) and specific breeds were not recorded. The sentence has been updated to accurately reflect data collected on each animal (line 69-70).
- Comment- Please discuss the level of experience of the observer who measured the alignments. Did the observer train to measure alignments before the study? (line 185).
- Response- An updated statement in the methods section is included to describe the training involved in taking radiographic measurements prior to the start of the study (line 94-96).
- Comment- You are talking about post-operative complications reported in the literature. Did you have any post-operative complications in included dogs? Please discuss it (line 158).
- Response- Additional sentences have been added that describe the number of dogs with post-operative patellar luxation and the associated grade of MPL for each dog (line 176-179).
- Comment- The sentence "another limitation of the study was" is incomplete, please delete it (line 190).
- Response- As suggested, this statement has been deleted.
