# Supplementary material for: Radiographic evaluation of patellar tendon length following corrective surgical procedures for medial patellar luxation in dogs
Source: PLoS One. 2020 Sep 4;15(9):e0238598. doi: 10.1371/journal.pone.0238598 (PMC7473547; doi:10.1371/journal.pone.0238598)
Supplement: Attachment — Submitted filename: suggested changes.docx [file pone.0238598.s003.docx]

Response to reviewers:

All suggested changes have been made.
